# Sparse time-varying log-ratios for longitudinal high-throughput sequencing data

**DOI:** 10.3389/fbinf.2026.1824265

**Published:** 2026-06-26

**Authors:** Ruijin Lu, Guoqi Yu, Cuilin Zhang, Zhen Chen

**Affiliations:** 1 Center for Biostatistics and Data Science, Washington University School of Medicine, St. Louis, MO, United States; 2 Global Center for Asian Women’s Health and Department of Obstetrics and Gynaecology, Yong Loo Lin School of Medicine, National University of Singapore, Singapore, Singapore; 3 Bia-Echo Asia Centre for Reproductive Longevity & Equality (ACRLE), Yong Loo Lin School of Medicine, National University of Singapore, Singapore, Singapore; 4 Eunice Kennedy Shriver National Institute of Child Health and Human Development, National Institutes of Health, Bethesda, MD, United States

**Keywords:** longitudinal omics data, log-ratio, compositional data, time-varying, variable selection

## Abstract

High-throughput, longitudinal omics data, such as metabolomics or microbiome profiles, present analytical challenges owing to their compositional nature and irregular observation times. Although existing approaches can address compositional or temporal aspects separately, very few are tailored to capture both properties simultaneously in a high-dimensional setting. We introduce LCoDaCoRe as a supervised learning method to identify sparse time-varying log-ratio features from longitudinal, compositional data. The proposed approach integrates functional data analysis and continuous relaxation to enable efficient feature selection from the log-ratio values. By expanding the log-transformed trajectories in their eigenspaces, LCoDaCoRe accommodates both dense and sparse sampling designs. In simulation studies, the proposed method demonstrated favorable performance in terms of predictive accuracy, selection sparsity, and precision compared to cross-sectional methods across varying correlation structures and outcome prevalence levels. Finally, we applied LCoDaCoRe to longitudinal lipidomics data from the NICHD Fetal Growth Studies and identified a highly interpretable log-ratio of triglycerides to sphingolipids that yielded more stable selection and better predictions for large-for-gestational-age births.

## 1 Introduction

The advent of high-throughput sequencing technologies has enabled the longitudinal profiling of complex biological systems, including metabolomics, lipidomics, and microbiome compositions. These data provide opportunities to study dynamic biological processes and have become increasingly prominent in biomedical research. For example, longitudinal lipidomics can reveal the gestational changes in lipid metabolism during pregnancy. However, analyzing these data poses several statistical challenges; the measurements are often high-dimensional and compositional, and the longitudinal design introduces within-subject correlations that can lead to misleading conclusions if ignored. In addition, repeated measurements are often obtained at irregular time points and may be sparsely observed across individuals, making it difficult to characterize trajectories consistently across the study cohort. These features necessitate modeling strategies that can jointly handle the compositional structure, temporal dynamics, and sparse sampling patterns of longitudinal data.

Many high-throughput omics data, including microbiome, metabolomics, and lipidomics profiles, are inherently compositional (Quinn et al., 2019), meaning that only the relative proportions of the components are informative while the total abundance is arbitrary. This is because the measurements often reflect constrained resources (e.g., total ion intensity, sequencing depth) or normalization procedures, which render the absolute values incomparable across samples. For example, in untargeted metabolomics, the measured peak intensities reflect signal abundances dependent on the total ion currents. As a result, two samples with the same absolute metabolite concentrations can still yield different raw intensities when analyzed on the same instrument under identical preparation procedures (Dunn et al., 2011). Thus, applying standard multivariate analyses directly to raw intensities or normalized abundances can yield misleading results due to the unit-sum constraint and induced negative correlations among the components. Hence, the log-ratio transformation has become a widely used strategy in compositional data analysis (Quinn et al., 2019; Aitchison, 1982). The log-ratio value expresses the relative abundances among components or groups of components and offers properties such as scale invariance and subcompositional coherence (Egozcue and Pawlowsky-Glahn, 2005); these features are biologically meaningful. Many established biomarkers, such as the plasma amyloid- β ratio in Alzheimer’s disease (Schultz et al., 2024) and the ratio of free triiodothyronine (fT3) to free thyroxine (fT4) in thyroid function assessment (Rawal et al., 2018), reflect contrasts between molecular groups. Therefore, modeling in the log-ratio space improves both statistical validity and biological interpretability.

Among log-ratio approaches, the balance feature, defined as the log-ratio between the geometric means of two disjoint groups of components, is appealing for its flexibility (Aitchison, 1982). It generalizes simple ratios by contrasting sets of components. Building on this idea, the compositional data via continuous relaxation (CoDaCoRe) framework (Gordon-Rodriguez et al., 2022) introduced a supervised learning approach that uses continuous relaxation to efficiently identify sparse and predictive log-ratios from cross-sectional data. However, CoDaCoRe and related approaches have not been developed for longitudinal compositional data, where the goal is to identify predictive log-ratio features whose trajectories, along with their effects on the outcome, can vary over time. Longitudinal compositional data are analytically challenging as they are no longer static compositions but trajectories that evolve over time and are often observed with sparse or irregular schedules. While the majority of longitudinal omics methods model compositional features as outcomes by either recovering trajectories from incomplete observations or performing featurewise inference for temporal associations and group differences using generalized mixed-effects, two-part, or spline-based frameworks (Peleg and Borenstein, 2024; Zhang and Yi, 2020; Shields-Cutler et al., 2018; Chen and Li, 2016; Metwally et al., 2018), some works have instead used longitudinal omics profiles as predictors of clinical outcomes. Some of these approaches reduce trajectories to scalar summaries before regression; for example, coda4microbiome uses area-under-the-curve (AUC) summaries of log-ratio trajectories, while PROLONG uses first differences in a penalized regression framework (Calle et al., 2023; Broll et al., 2025). Alternatively, Sun et al. (2020) proposed functional log-contrast regression for compositional trajectories. However, these methods do not address the supervised construction of sparse log-ratio features, such as balances, from a high-dimensional set of longitudinal compositional predictors.

In this study, we extended CoDaCoRe by introducing a supervised learning framework for longitudinal compositional data via continuous relaxation(LCoDaCoRe). Here, LCoDaCoRe represents log-transformed trajectories via functional principal component analysis (FPCA) and constructs interpretable log-ratio features (balances) whose values and associations with the outcomes can evolve over time. To identify sparse and predictive features, LCoDaCoRe employs a differentiable optimization routine as in CoDaCoRe and extends balance-based learning to longitudinal compositional settings. LCoDaCoRe uses FPCA to represent the longitudinal predictors via principal analysis by conditional expectation (PACE; Yao et al., 2005), which is designed for estimation with sparse and irregular observations. In the functional data analysis, repeated measurements are modeled as noisy observations from subject-specific realizations of a common smooth stochastic process over a continuous time domain (Morris, 2015; Ramsay and Dalzell, 1991). In our framework, we assumed that the log-transformed predictor trajectories shared a common basis representation so that each predictor trajectory could be expressed using the same set of basis functions with predictor- and subject-specific coefficients. Under this assumption, the induced log-ratio features (i.e., balances) can be represented using the same basis system; these representations reformulate the functional compositional problem in terms of the associated coefficients and yield a tractable multivariate formulation for balance learning.

We evaluated the performance of LCoDaCoRe through extensive simulations with varied correlation structures, signal sparsity, and case prevalence and benchmarked it against cross-sectional alternatives. We further applied our method to lipidomics data from the National Institute of Child Health and Human Development (NICHD) Fetal Growth Studies (Grewal et al., 2018; Louis et al., 2015) with the aim of identifying time-varying lipid signatures predictive of large-for-gestational-age (LGA) births. These analyses show that LCoDaCoRe meets our main goal of extending log-ratio learning to longitudinal compositional data, producing stable and interpretable features that improve both predictive accuracy and model parsimony. The remainder of this manuscript is organized as follows. Section 2 introduces the methodological components and statistical framework of LCoDaCoRe. Section 3.1 reports on our simulation studies evaluating the predictive accuracy, sparsity, and variable selection performances. Section 3.2 applies the proposed method to longitudinal lipidomics data from the NICHD Fetal Growth Studies cohort. Finally, Section 4 presents a discussion of the implications, limitations, and future directions of this approach.

## 2 Materials and methods

### 2.1 Problem formulation

We consider a supervised learning problem $x_{i}\left(t\right)\mapsto y_{i}$ , where the predictors $x_{i}\left(t\right)$ are longitudinal metabolomics or other compositional data at time t , and the response $y_{i}$ is an outcome of interest. Specifically, for a subject i , the expression

$$x_{i}\left(t\right)={\left[x_{i1}\left(t\right),\ldots ,x_{ip}\left(t\right)\right]}^{T}\in S^{p-1}$$

denotes the vector of compositions of p metabolites observed at time t . Here, $S^{p-1}=\left\{{\left[x_{1},\ldots ,x_{p}\right]}^{T}\in R^{p}:x_{j}>0,\;\sum_{j=1}^{p}x_{j}=1\right\}$ denotes the (p−1) -dimensional positive simplex in $R^{p}$ . The outcome $y_{i}$ can be either binary or continuous; for binary outcomes, it indicates whether the i th subject is a member of the case or non-case group; for continuous outcomes, $y_{i}$ represents a quantitative phenotype of interest. Our goal is to learn predictive features from the longitudinal compositional trajectories $x_{i}\left(t\right)$ associated with $y_{i}$ .

### 2.2 Background

#### 2.2.1 Log-ratio-transformed features

Compositionality implies that each element of $x_{i}\left(t\right)$ is inherently dependent on the others. To address this constraint, we construct log-ratio-transformed features that encode relative abundances in an unconstrained space where standard regression techniques are applicable. These log-ratio values can be formed between individual components or between aggregated groups using sums or geometric means. Their construction naturally ensures two key properties: scale invariance, meaning that the results are unaffected by the overall magnitude of a sample, and subcompositional coherence, where the interpretations remain valid even when the analysis is restricted to a subset of components. For example, the interpretation of the log-ratio $\log\left(x_{i1}/x_{i2}\right)$ remains the same regardless of whether the full composition $\left(x_{i1},\ldots ,x_{ip}\right)$ or just the subset $\left(x_{i1},x_{i2}\right)$ is analyzed. These properties are fundamental to compositional data analysis, where only relative information is meaningful (Aitchison, 1982). In addition to these statistical advantages, the log-ratio values capture molecular balances that better reflect physiological processes than absolute concentrations sometimes. For example, the total cholesterol to high-density lipoprotein cholesterol (HDL-C) ratio is an established indicator of lipid atherogenesis as it reflects the balance of cholesterol transport into and out of the arterial intima rather than the level of a single lipid fraction (Nam et al., 2006).

#### 2.2.2 Balances

One class of log-ratio values, known as balances (Egozcue and Pawlowsky-Glahn, 2005), has attracted attention in certain applications (Gordon-Rodriguez et al., 2022). A balance is defined as the log-ratio between the geometric means of two subsets of predictors, as shown in Equation 1:

$$\begin{array}{ll} B\left(x_{i};J^{+},J^{-}\right) & =\log\left\{\frac{{\left(\prod_{j\in J^{+}}x_{ij}\right)}^{\frac{1}{p^{+}}}}{{\left(\prod_{j\in J^{-}}x_{ij}\right)}^{\frac{1}{p^{-}}}}\right\}\\ & =\frac{1}{p^{+}}\underset{j\in J^{+}}{\sum }\log\left(x_{ij}\right)-\frac{1}{p^{-}}\underset{j\in J^{-}}{\sum }\log\left(x_{ij}\right), \end{array}$$ (1)

where $J^{+}$ and $J^{-}$ are disjoint subsets of the indices {1,…,p} , and $p^{+}$ and $p^{-}$ are their respective sizes. Balances generalize simple pairwise log-ratios (i.e., $p^{+}$ = $p^{-}$ = 1) by contrasting the geometric means of two groups of predictors. This broader formulation offers three key advantages. First, balances provide modeling flexibility by allowing multiple predictors to contribute to both the numerator and denominator, thereby expanding the space of admissible contrasts beyond single-pair comparisons. Second, they yield concise and biologically interpretable biomarkers to quantify the relative contributions of one group to another. Third, balances retain additivity in the logarithmic scale (Equation 1), which makes them directly compatible with regression models, thereby easing their extension to longitudinal data.

#### 2.2.3 Scalar-on-function regression

To model the time-varying features against a scalar outcome, we adopt a regression framework in which the predictor is a function and the outcome is a scalar. This falls within the scope of functional data analysis (Ramsay, 2005) and specifically the scalar-on-function regression, which characterizes the relationships between functional predictors and scalar responses. In this section, we briefly review some scalar-on-function regression models. For simplicity, we focus on the case of a univariate functional predictor $x_{i}\left(t\right)$ here, and the extension to a multivariate predictor $x_{i}\left(t\right)$ will be introduced in Section 2.3.

The majority of scalar-on-function methods are based on the functional linear model (FLM), which was first introduced by Ramsay and Dalzell (1991) and later formalized by Hastie and Mallows (1993) in the widely used form shown in Equation 2:

$$y_{i}=\beta_{0}+\int_{T}x_{i}\left(t\right)\beta \left(t\right)dt+\epsilon_{i},$$ (2)

where $y_{i}\in R$ is a continuous scalar response for subject i=1,…,N , $x_{i}\left(t\right)\in L^{2}\left(T\right)$ is a square-integrable functional predictor on the compact domain T⊂R , $\beta \left(t\right)\in L^{2}\left(T\right)$ is the coefficient function, $\beta_{0}$ is the intercept, and $\epsilon_{i}∼N\left(0,\sigma^{2}\right)$ are independent errors. The integral $\int_{T}x_{i}\left(t\right)\beta \left(t\right)dt$ represents the $L^{2}$ inner product between the predictor and coefficient functions. For non-Gaussian responses, this framework is extended to the generalized FLM introduced by Marx and Eilers (1999), which assumes that the conditional distribution of $y_{i}$ belongs to an exponential family and models the mean response through a link function g(⋅) given by Equation 3:

$$g\left(E\left(y_{i}\right)\right)=\beta_{0}+\int_{T}x_{i}\left(t\right)\beta \left(t\right)dt.$$ (3)

Since any square-integrable function in $L^{2}\left(T\right)$ allows basis expansion, we approximate the integral term in the FLM using truncated basis representations $x_{i}\left(t\right)=\sum_{k=1}^{K_{X}}x_{ik}^{*}\phi_{k}\left(t\right)$ and $\beta \left(t\right)=\sum_{\ell =1}^{K_{B}}\beta_{\ell }^{*}\psi_{\ell }\left(t\right)$ , where $\left\{\phi_{k}\left(t\right)\right\}$ and $\left\{\psi_{\ell }\left(t\right)\right\}$ are prespecified basis functions. Let $x_{i}^{*}={\left(x_{i1}^{*},\ldots ,x_{iK_{X}}^{*}\right)}^{⊤}$ , $\beta^{*}={\left(\beta_{1}^{*},\ldots ,\beta_{K_{B}}^{*}\right)}^{⊤}$ , $\phi \left(t\right)={\left(\phi_{1}\left(t\right),\ldots ,\phi_{K_{X}}\left(t\right)\right)}^{⊤}$ , and $\psi \left(t\right)={\left(\psi_{1}\left(t\right),\ldots ,\psi_{K_{B}}\left(t\right)\right)}^{⊤}$ ; then,

$$\int_{T}x_{i}\left(t\right)\beta \left(t\right)\;dt=x_{i}^{*⊤}J_{\phi ,\psi }\beta^{*}=x_{i}^{**⊤}\beta^{*},$$ (4)

In Equation 4, $J_{\phi ,\psi }=\int_{T}\phi \left(t\right)\psi {\left(t\right)}^{⊤}\;dt$ is the cross-inner-product matrix of the two basis systems and $x_{i}^{**⊤}=x_{i}^{*⊤}J_{\phi ,\psi }$ . This formulation reduces the functional linear model to a multiple linear regression in $\beta^{*}$ . In practice, this formulation allows two useful simplifications. First, if the same orthonormal basis is used for both the predictor and coefficient functions, then $J_{\phi ,\psi }=I$ . Second, if subjects are observed on a common grid $t=\left(t_{1},\ldots ,t_{T}\right)$ , then $J_{\phi ,\psi }$ can be approximated numerically using quadrature as $J_{\phi ,\psi }\approx \Phi W\Psi^{⊤}$ , where ${\left(\Phi \right)}_{k,r}=\phi_{k}\left(t_{r}\right)$ , ${\left(\Psi \right)}_{\ell ,r}=\psi_{\ell }\left(t_{r}\right)$ , and $W=diag\left(w_{1},\ldots ,w_{T}\right)$ contains the quadrature weights. For an equally spaced grid, this reduces to $J_{\phi ,\psi }\approx \Delta t\;\Phi \Psi^{⊤}$ .

One common choice of basis can be obtained using the functional principal components (fPCs) that are estimated from the eigen decomposition of the covariance surface of the predictor functions (Ramsay and Silverman, 2005). The fPCs form an orthonormal basis for $L^{2}\left(T\right)$ and are naturally ordered by decreasing eigenvalues. Regularization can be achieved by truncating the expansion and retaining only the leading components that capture a large percentage of the total variability (Cardot et al., 1999; Müller and Stadtmüller, 2005). For irregular and sparse functions, Yao et al. (2005) proposed the principal analysis by conditional expectation (PACE) method that borrows information across all curves using a kernel-smoothed covariance matrix to estimate the eigenfunctions.

#### 2.2.4 FPCA for sparse data: PACE

In longitudinal study designs, the subjects are observed only a few times and at irregular time points. PACE (Yao et al., 2005) addresses this challenge by estimating the subject-specific fPC scores using both individual-level observations and information pooled across all subjects. This enables fPC expansion to be used in downstream analyses, such as scalar-on-function regression. Consistent with Equation 2, the univariate predictor trajectory for subject i is denoted as $x_{i}\left(t\right)$ . Here, we model it as an independent realization of a smooth random function X(t) , with mean function μ(t)=E[X(t)] and covariance function G(s,t)=cov(X(s),X(t)) . Under standard assumptions, the covariance function can be represented by the Karhunen–Loève expansion as $\;G\left(s,t\right)=\sum_{k=1}^{\infty }\lambda_{k}\phi_{k}\left(s\right)\phi_{k}\left(t\right)$ , where $\left\{\phi_{k}\right\}$ are orthonormal eigenfunctions in $L^{2}\left(T\right)$ and $\left\{\lambda_{k}\right\}$ are the corresponding non-increasing eigenvalues. Then, $x_{i}\left(t\right)$ can be expressed as Equation 5:

$$x_{i}\left(t\right)=\mu \left(t\right)+\overset{\infty }{\underset{k=1}{\sum }}\xi_{ik}\phi_{k}\left(t\right),t_{ij}\in T,$$ (5)

where $\xi_{ik}=\int \left(x_{i}\left(t\right)-\mu \left(t\right)\right)\phi_{k}\left(t\right)dt$ are the fPC scores.

Let $x_{i}=\left(x_{i1},\ldots ,x_{iT_{i}}\right)$ and $\mu_{i}=\left(\mu \left(t_{i1}\right),\ldots ,\mu \left(t_{iT_{i}}\right)\right)$ denote the observed data and mean vector for subject i . These observed data are subject to measurement errors. The j th observation from subject i , at time $t_{ij}$ for $j=1,\ldots ,T_{i}$ is modeled as $x_{ij}=x_{i}\left(t_{ij}\right)+\epsilon_{ij}$ , where $\epsilon_{ij}$ are independent and identically distributed measurement errors with mean 0 and variance $\sigma^{2}$ . The number of measurements obtained from the i th subject, $T_{i}$ , can vary, reflecting the irregular sampling times. PACE first estimates the mean function μ(t) and the covariance function G(s,t) as $\hat{\mu }\left(t\right)$ and $\hat{G}\left(s,t\right)$ , respectively, from the pooled observations of all subjects. The eigen decomposition of the smoothed covariance function is then performed to obtain the estimated eigenfunctions $\hat{\phi_{k}}$ and eigenvalues ${\hat{\lambda }}_{k}$ . The measurement error $\sigma^{2}$ is estimated as the average difference between the diagonal of the estimated G(s,t) and a local linear smoother focusing on the diagonal values. Given these estimates, the fPC score $\xi_{ik}$ for each subject is predicted by conditional expectation under a joint Gaussian assumption as

$${\hat{\xi }}_{ik}={\hat{\lambda }}_{k}{\hat{\phi }}_{ik}^{T}{\hat{\Sigma_{i}}}^{-1}\left(x_{i}-{\hat{\mu }}_{i}\right),$$

where ${\hat{\phi }}_{ik}={\left({\hat{\phi }}_{k}\left(t_{i1}\right),\ldots ,{\hat{\phi }}_{k}\left(t_{iT_{i}}\right)\right)}^{T}$ , ${\left({\hat{\Sigma }}_{i}\right)}_{j,l}=\hat{G}\left(t_{ij},t_{il}\right)+{\hat{\sigma }}^{2}\delta_{jl}$ , and ${\hat{\mu }}_{i}=\left(\hat{\mu }\left(t_{i1}\right),\ldots ,\hat{\mu }\left(t_{iT_{i}}\right)\right)$ . Suppose we retain the first K fPCs, $x_{i}^{**}$ in Equation 4 can be replaced by $\left(\hat{\xi_{i1}},\ldots ,\hat{\xi_{iK}}\right)$ to estimate $\beta^{*}$ . We denote the estimated $\beta^{*}$ as ${\hat{\beta }}^{*}=\left({\hat{\beta }}_{1}^{*},\ldots ,{\hat{\beta }}_{K}^{*}\right)$ . These enable us to reconstruct and estimate the coefficient function in the time domain through $\sum_{k}\hat{\beta_{k}^{*}}{\hat{\phi }}_{k}\left(t\right)$ . The estimation details can be found in Yao et al. (2005).

### 2.3 Methods

We now present a novel supervised learning algorithm for longitudinal compositional data via continuous relaxation (LCoDaCoRe).

#### 2.3.1 Model

Assume that the log-ratio-transformed features, specifically the balances, have time-homogeneous compositions, i.e., the compositions of the numerator and denominator of each feature do not change over time. We define the time-varying balance B(t) as Equation 6, as follows:

$$\begin{array}{ll} B\left(x_{i}\left(t\right),J^{+},J^{-}\right) & =log\left\{\frac{{\left(\prod_{j\in J^{+}}x_{ij}\left(t\right)\right)}^{1/p^{+}}}{{\left(\prod_{j\in J^{-}}x_{ij}\left(t\right)\right)}^{1/p^{-}}}\right\}\\ & =\frac{1}{p^{+}}\underset{j\in J^{+}}{\sum }log\left(x_{ij}\left(t\right)\right)-\frac{1}{p^{-}}\underset{j\in J^{-}}{\sum }log\left(x_{ij}\left(t\right)\right), \end{array}$$ (6)

where $x_{ij}\left(t\right)$ is the j th predictor of the i th subject at time t with $\sum_{j}x_{ij}\left(t\right)=1$ for any i∈{1,…,N} and t∈T ; $J^{+}$ and $J^{-}$ that are not indexed using t , are disjoint subsets of the predictor index set {1,…,p} with sizes $p^{+}$ and $p^{-}$ , respectively.

The functional balances defined above are then passed to the downstream generalized regression model as

$$g\left(E\left(y_{i}\right)\right)=f\left(x_{i}\left(t\right)\right)=\alpha +\int \beta \left(t\right)\cdot B\left(x_{i}\left(t\right);J^{+},J^{-}\right)dt,$$

where $B\left(x_{i}\left(t\right);J^{+},J^{-}\right)$ denotes the balance as a function of t (t∈T) , α is a scalar, and β(t) is a coefficient function of t . For clarity, we restrict our formulation to detecting one balance feature at a time. In practice, the algorithm repeats the regression step and updates the residuals so that multiple balances can be identified sequentially. The framework can also be extended to non-linear associations as long as they are differentiable.

To facilitate estimation, we introduce the log-transformed composition vector for subject i as

$$z_{i}\left(t\right)={\left[z_{i1}\left(t\right),\ldots ,z_{ip}\left(t\right)\right]}^{T},\;z_{ij}\left(t\right)=log\left(x_{ij}\left(t\right)\right),$$

which provides a direct representation of the predictors on the log scale. The time-varying function in Equation 6 can be expressed equivalently as Equation 7:

$$B\left(z_{i}\left(t\right);J^{+},J^{-}\right)=\frac{1}{p^{+}}\underset{j\in J^{+}}{\sum }z_{ij}\left(t\right)-\frac{1}{p^{-}}\underset{j\in J^{-}}{\sum }z_{ij}\left(t\right).$$ (7)

When the collection times are sparsely and irregularly sampled across subjects, we represent each log-transformed trajectory through the PACE framework. Let the discrete noisy observations of predictor j for subject i be denoted by $z_{ij}=\left(z_{\mathrm{ij1}},\ldots ,z_{ijT_{i}}\right)$ , where $z_{\mathrm{ijl}}=z_{ij}\left(t_{l}\right)+\epsilon_{\mathrm{ijl}}\;\left(l=1,\ldots ,T_{i}\right)$ . We treat the latent trajectory $z_{ij}\left(t\right)$ as an independent realization of a smooth random function $Z_{j}\left(t\right)$ with mean function $\mu_{j}\left(t\right)=E\left\{Z_{j}\left(t\right)\right\}$ and covariance function $G_{j}\left(s,t\right)=cov\left\{Z_{j}\left(s\right),Z_{j}\left(t\right)\right\}$ . By the Karhunen–Loève expansion,

$$z_{ij}\left(t\right)=\mu_{j}\left(t\right)+\overset{\infty }{\underset{k=1}{\sum }}\xi_{\mathrm{ijk}}\phi_{k}\left(t\right),$$

where $\xi_{\mathrm{ijk}}$ are the fPC scores. We assume a common set of eigenfunctions $\left\{\phi_{k}\left(t\right)\right\}$ across the predictors so that the basis domain is shared when constructing the balances. To estimate this shared basis, we pool the log-transformed predictor trajectories $\left\{z_{ij}\left(t\right):i=1,\ldots ,n,\;j=1,\ldots ,p\right\}$ across both subjects and predictors and apply the FPCA via the PACE algorithm to the pooled trajectories. The resulting pooled covariance estimate is eigen-decomposed to obtain the shared eigenfunctions $\left\{{\hat{\phi }}_{k}\left(t\right)\right\}$ , and ${\hat{\xi }}_{\mathrm{ijk}}$ denotes the corresponding score for predictor j in subject i . Following the estimation steps in Section 2.2.4, each trajectory is approximated by its projection onto the space spanned by the first K estimated eigenfunctions as

$${\hat{z}}_{ij}^{K}\left(t\right)={\hat{\mu }}_{j}\left(t\right)+\overset{K}{\underset{k=1}{\sum }}{\hat{\xi }}_{\mathrm{ijk}}{\hat{\phi }}_{k}\left(t\right),$$

where ${\hat{\mu }}_{j}\left(t\right)$ , ${\hat{\xi }}_{\mathrm{ijk}}$ , and ${\hat{\phi }}_{k}\left(t\right)$ denote the estimated mean function, fPC scores, and eigenfunctions, respectively.

Correspondingly, given two disjoint subsets $J^{+}$ and $J^{-}$ in {1,…,p} , the balance is obtained as Equation 8:

$$\begin{array}{ll} B\left({\hat{z}}_{i}^{K}\left(t\right),J^{+},J^{-}\right) & =\frac{1}{p^{+}}\underset{j\in J^{+}}{\sum }{\hat{z}}_{ij}^{K}\left(t\right)-\frac{1}{p^{-}}\underset{j\in J^{-}}{\sum }{\hat{z}}_{ij}^{K}\left(t\right)\\ & =\Delta {\hat{\mu }}_{J^{+},J^{-}}\left(t\right)+\frac{1}{p^{+}}\underset{j\in J^{+}}{\sum }\overset{K}{\underset{k=1}{\sum }}{\hat{\xi }}_{\mathrm{ijk}}{\hat{\phi }}_{k}\left(t\right)-\frac{1}{p^{-}}\underset{j\in J^{-}}{\sum }\overset{K}{\underset{k=1}{\sum }}{\hat{\xi }}_{\mathrm{ijk}}{\hat{\phi }}_{k}\left(t\right)\\ & =\Delta {\hat{\mu }}_{J^{+},J^{-}}\left(t\right)+\overset{K}{\underset{k=1}{\sum }}B_{ik}^{*}\left({\hat{\xi }}_{ik},J^{+},J^{-}\right){\hat{\phi }}_{k}\left(t\right)\\ & =\Delta {\hat{\mu }}_{J^{+},J^{-}}\left(t\right)+B_{i}^{*}\left({\hat{\Xi }}_{i}^{K},J^{+},J^{-}\right)\hat{\phi }\left(t\right), \end{array}$$ (8)

where $\Delta {\hat{\mu }}_{J^{+},J^{-}}\left(t\right)=\frac{1}{p^{+}}\sum_{j\in J^{+}}{\hat{\mu }}_{j}\left(t\right)-\frac{1}{p^{-}}\sum_{j\in J^{-}}{\hat{\mu }}_{j}\left(t\right)$ , ${\hat{z}}_{i}^{K}\left(t\right)={\left({\hat{z}}_{i1}^{K}\left(t\right),\ldots ,{\hat{z}}_{ip}^{K}\left(t\right)\right)}^{T}$ , ${\hat{\xi }}_{ik}={\left({\hat{\xi }}_{\mathrm{i1k}},\ldots ,{\hat{\xi }}_{\mathrm{ipk}}\right)}^{T}$ , and ${\hat{\Xi }}_{i}^{K}=\left({\hat{\xi }}_{i1},\ldots {\hat{\xi }}_{iK}\right)$ . Here, $B_{i}^{*}\left({\hat{\Xi }}_{i}^{K},J^{+},J^{-}\right)$ is a K -dimensional coefficient vector representing the balance in the basis domain for subject i . Equation 8 shows that the balance can be expanded using the same set of eigenfunctions, where the coefficients are given by the contrasts of the fPC scores averaged over the log-transformed predictors in $J^{+}$ and $J^{-}$ . Suppose we expand the coefficient function β(t) using the same basis, i.e., $\beta \left(t\right)=\sum_{k=1}^{K}\beta_{k}^{*}{\hat{\phi }}_{k}\left(t\right)$ ; as presented in the discussion following Equation 4, the cross-basis transformation reduces to the identity matrix. Hence, we can rewrite the regression model in the simplified vector form as Equation 9:

$$f\left(x_{i}\left(t\right)\right)=\tilde{\alpha }+B_{i}^{*}\left({\hat{\Xi }}_{i}^{K},J^{+},J^{-}\right)\beta^{*},$$ (9)

where $\tilde{\alpha }=\alpha +\int \beta \left(t\right)\Delta {\hat{\mu }}_{J^{+},J^{-}}\left(t\right)dt$ is the reparametrized intercept that absorbs the deterministic mean-contrast term, and $\beta^{*}=\left(\beta_{1}^{*},\ldots ,\beta_{K}^{*}\right)$ . Given this simplified model, our goal is to estimate the regression parameters $\left(\tilde{\alpha },\beta^{*}\right)$ and the compositions of the balance $\left(J^{-},J^{+}\right)$ . In the next section, we introduce a method to estimate these simultaneously through continuous relaxation at a reasonable computational cost.

#### 2.3.2 Estimation via continuous relaxation

The outcome is modeled through a generalized linear model framework $g\left(E\left(y_{i}\right)\right)=f\left(x_{i}\left(t\right)\right)=\tilde{\alpha }+B_{i}^{*}\left({\hat{\Xi }}_{i}^{K},J^{+},J^{-}\right)\beta^{*}$ , where g(⋅) is a link function (e.g., logit for binary outcomes). We estimate $\left(\tilde{\alpha },\beta^{*},J^{+},J^{-}\right)$ by maximizing the likelihood function or equivalently by minimizing the negative log-likelihood loss, which we define as $L\left(y_{i},f\left(x_{i}\left(t\right)\right)\right)$ . However, identifying the balance requires selection of a discrete subset over $\left(J^{+},J^{-}\right)$ . Following the method by Gordon-Rodriguez et al. (2022), we approximate this discrete selection through continuous relaxation, which replaces the hard assignments of predictors to numerator or denominator groups with soft assignments. This relaxation yields a smooth optimization problem that can be solved efficiently by gradient descent algorithms.

In continuous relaxation, each predictor (e.g., the trajectory of a metabolite) is assigned an unconstrained weight. Let the weight vector be $\omega =\left(\omega_{1},\ldots ,\omega_{p}\right)\in R$ . These weights are mapped to soft assignments by applying the sigmoid transformation in Equation 10:

$$\tilde{\omega }=2\cdot sigmoid\left(\omega \right)-1=\frac{2}{1+exp\left(-\omega \right)}-1,$$ (10)

This maps each $\omega_{j}$ into the interval (−1,1) . This mapping serves as a smooth relaxation of the discrete set {−1,1,0} corresponding to membership in $J^{-}$ , $J^{+}$ , and exclusion, respectively. We define ${\tilde{\omega }}^{+}=ReLU\left(\tilde{\omega }\right)$ and ${\tilde{\omega }}^{-}=ReLU\left(-\tilde{\omega }\right)$ , where ReLU(ω)=max(ω,0) is applied componentwise. The balance function in Equation 1 is then approximated by Equation 11:

$$\begin{array}{ll} \tilde{B}\left(x_{i}\left(t\right),\omega \right) & =\frac{\sum_{j}{\tilde{\omega }}_{j}^{+}\log x_{ij}\left(t\right)}{\sum_{j}{\tilde{\omega }}_{j}^{+}}-\frac{\sum_{j}{\tilde{\omega }}_{j}^{-}\log x_{ij}\left(t\right)}{\sum_{j}{\tilde{\omega }}_{j}^{-}}\\ & =\frac{\langle {\tilde{\omega }}^{+},\log x_{i}\left(t\right)\rangle }{\|{\tilde{\omega }}^{+}{\|}_{1}}-\frac{\langle {\tilde{\omega }}^{-},\log x_{i}\left(t\right)\rangle }{\|{\tilde{\omega }}^{-}{\|}_{1}}. \end{array}$$ (11)

Because ReLU is not differentiable at zero and Equation 11 is undefined when either $\|{\tilde{\omega }}^{+}{\|}_{1}=0$ or $\|{\tilde{\omega }}^{-}{\|}_{1}=0$ , the resulting surrogate objective is not globally smooth on $R^{p}$ . Instead, optimization is carried out over the domain

$$\Omega =\left\{\omega \in R^{p}:\|{\tilde{\omega }}^{+}{\|}_{1}>0,\|{\tilde{\omega }}^{-}{\|}_{1}>0\right\},$$

on which the objective is well-defined and almost everywhere differentiable. In other words, the subset-based geometric means are replaced by weighted geometric averages over all predictors. In the basis space, we can approximate the balance defined in Equation 8 as

$$\begin{array}{ll} & {\tilde{B}}^{K}\left({\hat{z}}_{i}^{K}\left(t\right),\omega \right)=\frac{\sum_{j}{\tilde{\omega }}_{j}^{+}{\hat{z}}_{ij}^{K}\left(t\right)}{\sum_{j}{\tilde{\omega }}_{j}^{+}}-\frac{\sum_{j}{\tilde{\omega }}_{j}^{-}{\hat{z}}_{ij}^{K}\left(t\right)}{\sum_{j}{\tilde{\omega }}_{j}^{-}}\\ & \;=\frac{1}{\|{\tilde{\omega }}^{+}{\|}_{1}}\left({\tilde{\omega }}_{1}^{+},\ldots ,{\tilde{\omega }}_{p}^{+}\right)\left(\begin{array}{ccc} {\hat{\xi }}_{\mathrm{i11}} & \ldots & {\hat{\xi }}_{\mathrm{i1K}}\\ \vdots & \ddots & \vdots \\ {\hat{\xi }}_{\mathrm{ip1}} & \ldots & {\hat{\xi }}_{\mathrm{ipK}} \end{array}\right)\left(\begin{array}{c} {\hat{\phi }}_{1}\left(t\right)\\ \vdots \\ {\hat{\phi }}_{K}\left(t\right) \end{array}\right)\\ & \;-\frac{1}{\|{\tilde{\omega }}^{-}{\|}_{1}}\left({\tilde{\omega }}_{1}^{-},\ldots ,{\tilde{\omega }}_{p}^{-}\right)\left(\begin{array}{ccc} {\hat{\xi }}_{\mathrm{i11}} & \ldots & {\hat{\xi }}_{\mathrm{i1K}}\\ \vdots & \ddots & \vdots \\ {\hat{\xi }}_{\mathrm{ip1}} & \ldots & {\hat{\xi }}_{\mathrm{ipK}} \end{array}\right)\left(\begin{array}{c} {\hat{\phi }}_{1}\left(t\right)\\ \vdots \\ {\hat{\phi }}_{K}\left(t\right) \end{array}\right)\\ & \;={\tilde{B}}_{i}^{*}\left({\hat{\Xi }}_{i}^{K},\omega \right)\hat{\phi }\left(t\right). \end{array}$$

This relaxation is differentiable with respect to ω . As a result, we can estimate the parameters by minimizing a smooth surrogate objective jointly with respect to $\left(\omega ,\tilde{\alpha },\beta^{*}\right)$ through gradient descent as

$$\min_{\left(\omega ,\alpha ,\beta^{*}\right)}\underset{i}{\sum }L\left(y_{i},\tilde{\alpha }+{\tilde{B}}_{i}^{*}\left({\hat{\Xi }}_{i}^{K},\omega \right)\beta^{*}\right).$$

#### 2.3.3 Discretization and regularization

Although the relaxed balance in Equation 11 retains all the information required to minimize the objective function, it includes contributions from all the predictors and is difficult to interpret. Here, it is preferable to construct the balances from a small subset of variables for interpretability. To induce sparsity, we follow the method of Gordon-Rodriguez et al. (2022) and discretize the soft assignment vector $\tilde{\omega }$ by thresholding. For a fixed τ∈(0,1) , predictors are assigned to the subsets as

$${\tilde{J}}^{+}=\left\{j:{\tilde{\omega }}_{j}>\tau \right\},{\tilde{J}}^{-}=\left\{j:{\tilde{\omega }}_{j}<-\tau \right\}.$$

Given a trained $\tilde{\omega }$ and threshold τ , the corresponding balance is evaluated in the basis domain as shown in Equation 8, while the regression coefficients are estimated by refitting the regression model. Computationally, fitting a linear model is a very fast process, and this step can be repeated for a range of values of *τ* τ within a cross-validation framework to select the threshold $\hat{\tau }$ that optimizes predictive performance. Finally, the trained regression function is given by Equation 12:

$$\hat{f}\left(x_{i}\left(t\right)\right)=\hat{\tilde{\alpha }}+B_{i}^{*}\left({\hat{\Xi }}_{i}^{K},{\hat{J}}^{+},{\hat{J}}^{-}\right){\hat{\beta }}^{*},$$ (12)

where $\left({\hat{J}}^{+},{\hat{J}}^{-}\right)$ are the subsets corresponding to the optimal threshold $\hat{\tau }$ , and $\left(\hat{\tilde{\alpha }},{\hat{\beta }}^{*}\right)$ are the coefficients obtained by refitting the model on the selected subsets.

A larger τ will allow for the selection of fewer predictors, resulting in a sparser model. To choose the optimal threshold $\hat{\tau }$ , we apply the λ -standard-error rule, which picks the sparsest model with a mean cross-validated score within the λ standard errors of the optimum. In practice, we report results for λ=0 (no regularization) and λ=1 , which is the commonly used 1-standard-error rule (Hastie et al., 2009).

#### 2.3.4 Selection of the number of basis functions

The number of basis functions K used to represent the trajectories can be chosen in two ways. First, an information-loss threshold δ can be specified, and the smallest K is retained such that the first K eigenfunctions explain at least 1−δ of the total variation. Second, a cross-validation strategy can be used in which a large upper bound $K_{\max}$ is set, and the algorithm is implemented for each $K\in \left\{1,\ldots ,K_{\max}\right\}$ to select the value of K that optimizes predictive performance. The first approach is computationally efficient while the second approach is more adaptive. Our simulation studies (Section 3) show that both methods yield comparable results in terms of prediction accuracy and model sparsity.

#### 2.3.5 LCoDaCoRe algorithm

The full procedure of LCoDaCoRe is summarized in Algorithm 1, and the major steps are as follows:

Step 1: Basis construction. The shared eigenbasis is estimated by pooling the log-transformed predictor trajectories across subjects and predictors before applying FPCA via the PACE algorithm to the pooled trajectories.
Step 2: Basis selection. A truncated set of eigenfunctions is retained according to one of the strategies described in Section 2.3.4. The time-domain predictors are also represented in the reduced basis domain.
Step 3: Balance identification. A first balance is selected by fitting a regressor of the form shown in Equation 12.
Step 4: Sequential balance selection. At each subsequent step, a new balance is identified using a stagewise update conditional on the current model. Here, the fitted value from the previous step is carried forward as an offset in the linear predictor, and the new balance is estimated while treating this offset as fixed. For continuous outcomes, the offset is the current fitted mean. For binary outcomes, the offset is the current fitted value on the logit scale. The model is then updated by adding the fitted value of the new balance to the current fitted value. This procedure continues until the cross-validated performance (e.g., out-of-sample AUC or RMSE) does not improve. Multiple balances are identified sequentially and are ordered by their predictive contributions.

Algorithm 1 **LCoDaCoRe**

**Inputs:** Data ${\left(x_{i1},\ldots ,x_{it_{T_{i}}},y_{i}\right)}_{i\in \left\{1,\ldots ,N\right\}}$ ; index set of training data Tr ; basis selection method Model ; minimum information loss δ ; maximum number of regressors M

*# FPCA decomposition*
 Apply PACE to the pooled trajectories $\left\{\log x_{ij}\left(t\right):i=1,\ldots ,n;\;j=1,\ldots ,p\right\}.$

**if** Model == 1 **then**
 Truncate the number of basis functions by retaining 1−δ of the total information.
**else if** Model == 2 **then**
 Use cross-validation to find the optimal number of basis functions.
**end if**
Save the basis coefficients ${\left({\hat{\Xi }}_{i}^{K}\right)}_{i=1}^{n}$ and the basis functions $\hat{\phi }\left(t\right).$

*# Initialize the fitted value and iteration counter*
Take the training data ${\left({\hat{\Xi }}_{i}^{K}\right)}_{i\in Tr}$

Initialize a vector for the fitted values ${\hat{g}}^{\left(0\right)}=0.$

Initialize m=0 .
*# Main algorithm (train on training data)*
**while** m < M **do**
 Initialize a new relaxation ( ω , $\tilde{\alpha }$ , $\beta^{*}$ ).
 Train ( ω , $\tilde{\alpha }$ , $\beta^{*}$ ) by the stochastic gradient descent algorithm.
 Use cross-validation to find the optimal threshold $\hat{\tau }$ .
 Retrain ( $\tilde{\alpha }$ , $\beta^{*}$ ) using $\left({\hat{J}}^{+},{\hat{J}}^{-}\right)$ .
 Update the fitted value as ${\hat{g_{i}}}^{\left(m+1\right)}\leftarrow {\hat{g_{i}}}^{\left(m\right)}+{\hat{f}}_{m+1}\left(x_{i}\left(t\right)\right).$

 Save the estimated results ( $\hat{\tilde{\alpha }}$ , ${\hat{\beta }}^{*},\left({\hat{J}}^{+},{\hat{J}}^{-}\right)$ ) in the ensemble.
 **if** ${\hat{J}}^{+}=\emptyset$ or ${\hat{J}}^{-}=\emptyset$ **then**
  Break.
 **else**
  
m←m+1.

 **end if**
**end while**
**Return:** Basis functions $\left(\hat{\phi }\left(t\right)\right)$ ; estimated ensemble; iteration counter m ; fitted value $\hat{g}\left(x\right).$

## 3 Results

### 3.1 Simulation studies

#### 3.1.1 Simulation setup

**FIGURE 1:**
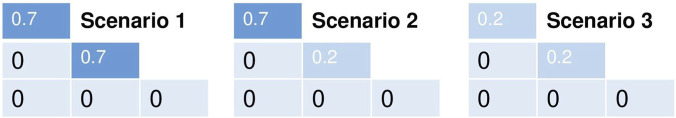
Correlation coefficients $\left(\rho_{Z}\right)$ between the predictors in the simulation study. The off-diagonal zeros indicate independent clusters, while the within-cluster correlations are shown along the diagonal entries.

To mimic the NICHD Fetal Growth Studies, we assumed four planned visits in the simulations that were spaced equally over [0,1], where each subject i∈{1,…,n} was observed for $T_{i}\in \left\{2,3,4\right\}$ randomly chosen visits, yielding irregular visit times $0\leq t_{1}<\ldots <t_{T_{i}}\leq 1$ . The within-subject temporal correlation was modeled by an $L_{1}$ -distance-based structure given by $\Sigma_{T}={\left[\rho_{T}^{\left|t_{s}-t_{s^{\prime }}\right|}\right]}_{T_{i}\times T_{i}}$ $\left(1\leq s,s^{\prime }\leq T_{i}\right)$ , with $\rho_{T}=0.4$ . The between-predictor correlation was modeled using a block diagonal matrix $\Sigma_{Z}={\left[\rho_{Z}^{I\left(j\neq j^{\prime }\right)}\right]}_{p\times p}$ with the predictors being grouped into three clusters: two active clusters having non-zero associations with the outcome and a null cluster having zero association with the outcome. The null cluster was uncorrelated $\left(\rho_{Z}=0\right)$ , while the two active clusters had within-cluster correlations according to one of the following three scenarios (Figure 1): both active clusters were strongly correlated $\left(\rho_{Z}=0.7\right)$ ; one cluster was strongly correlated $\left(\rho_{Z}=0.7\right)$ and the other was weakly correlated $\left(\rho_{Z}=0.2\right)$ ; both active clusters were weakly correlated $\left(\rho_{Z}=0.2\right)$ .

For each subject i , we generated $z_{i}$ from $N_{T_{i}p}\left(1_{T_{i}}⊗\mu ,\sigma_{Z}^{2}\left(\Sigma_{T}⊗\Sigma_{Z}\right)\right)$ , where $\mu ={\left(\mu_{1},\ldots ,\mu_{p}\right)}^{⊤}$ , $\sigma_{Z}=0.5$ , and $\mu_{j}\overset{\text{iid}}{∼}N\left(10.76,\;1.95\right)$ , reflecting the empirical distribution of the log-transformed real data. The compositional data were obtained by normalization as $x_{ij}\left(t_{is}\right)=\exp\left(z_{ij}\left(t_{is}\right)\right)/\sum_{j=1}^{p}\exp\left(z_{ij}\left(t_{is}\right)\right)$ . We set the sample size to n=100 and considered that the predictor dimensions p∈{30,100,200} represented the low-, moderate-, and high-dimensional settings, with the corresponding cluster sizes being [5, 6, 19], [15, 15, 70], and [30, 30, 140], respectively. To generate the balances and their basis-domain representations $B_{i}^{*}\left({\hat{\Xi }}_{i}^{K},J^{+},J^{-}\right)$ (Equation 8), we set the number of active predictors to [3, 3, 0] for p=30 and [5, 5, 0] for p=100 and 200. The binary outcomes were generated from the logistic regression model given by $logit\left\{\left(E\left(y_{i}\right)\right.\right\}=\alpha +B_{i}^{*}\left({\hat{\Xi }}_{i}^{K},J^{+},J^{-}\right)\beta^{*}$ , with intercept α=1 and coefficients $\beta^{*}={\left(2,0,\ldots ,0\right)}^{⊤}$ ; here the first coefficient was 2 while the remaining K−1 coefficients were 0. Because the prevalence was sensitive to the mean vector μ used to generate the “non-normalized” predictors, we screened 200 random seeds and selected three that yielded approximately 10%, 30%, and 50% case prevalences to represent the highly imbalanced, moderately imbalanced, and balanced scenarios.

Next, two versions of LCoDaCoRe were implemented. In LCoDaCoRe1, we selected the number of basis functions through 10-fold cross-validation by maximizing the out-of-sample AUC. In LCoDaCoRe2, we selected the smallest number of basis functions such that the root of the total squared reconstruction error of the trajectories was below $10^{-4}$ . As baseline comparisons, four cross-sectional CoDaCoRe models were fitted to construct the log-ratio features, where each model used snapshot data from one of the four visits. This design mirrored the motivating data, in which maternal blood samples were collected at the four visits and used to predict the fetal LGA values separately. Each simulation scenario was replicated 200 times, and a testing dataset of size $n_{te}=50$ was generated in each run. We compared the proposed and baseline approaches on three aspects: predictive accuracy, sparsity, and false negative rate (FNR). Predictive accuracy was measured by the AUC on the testing dataset, while sparsity was measured by the number of selected predictors, and FNR was measured by the proportion of truly active predictors missed. We selected the sparsity threshold $\hat{\tau }$ using the 1-standard-error rule, i.e., ${\hat{\tau }}_{\mathrm{1SE}}=\max\left\{\tau :\;L_{CV}\left(\tau \right)\leq \min_{\tau^{\prime }}L_{CV}\left(\tau^{\prime }\right)+SE_{\min}\right\}$ , where $L_{CV}\left(\tau \right)$ denotes the mean 10-fold cross-validated loss at threshold τ , 1−AUC for binary outcomes and RMSE for continuous outcomes, and $SE_{\min}$ is the standard error of the cross-validated loss at the threshold that achieves the minimum mean loss.

#### 3.1.2 Simulation results

**FIGURE 2:**
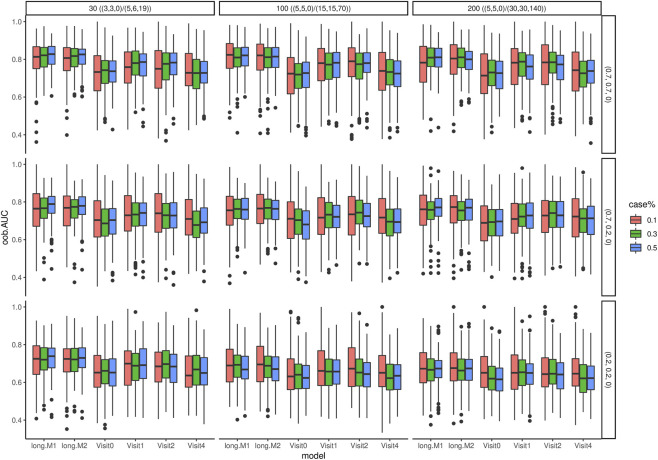
Boxplots of the out-of-sample area-under-the-curve (AUC) values based on 200 simulation replicates. Each block corresponds to a simulation scenario defined by the total number of predictors (column) and the within-cluster correlation structure (row). The column headings indicate the total number of predictors, and the values in the parentheses indicate the cluster-specific numbers of active/total predictors; the rows represent within-cluster correlation scenarios, with the headings specifying the correlation coefficients for the three clusters. The box colors denote outcome prevalence; within each cell, the results are shown for the two model variants (long.M1 for LCoDaCoRe1 and long.M2 for LCoDaCoRe2), and for the cross-sectional CoDaCoRe applied at each visit (0, 1, 2, 4).

**FIGURE 3:**
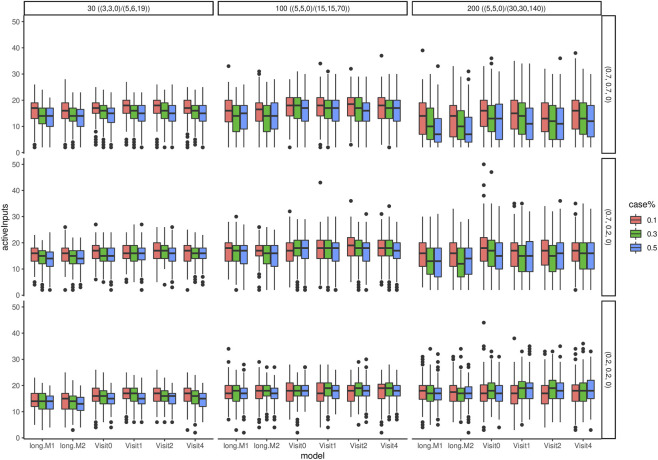
Boxplots of the numbers of selected predictors based on 200 simulation replicates. Each block corresponds to a simulation scenario defined by the total number of predictors (column) and the within-cluster correlation structure (row). The column headings indicate the total number of predictors, and the values in the parentheses indicate the cluster-specific numbers of active/total predictors; the rows represent within-cluster correlation scenarios, with the headings specifying the correlation coefficients for the three clusters. The box colors denote outcome prevalence; within each cell, the results are shown for the two model variants (long.M1 for LCoDaCoRe1 and long.M2 for LCoDaCoRe2), and for the cross-sectional CoDaCoRe applied at each visit (0, 1, 2, 4).

**FIGURE 4:**
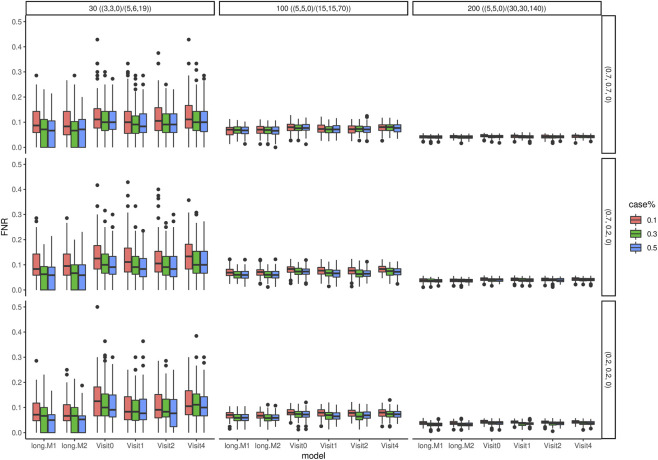
Boxplots of the false negative rate (FNR) values based on 200 simulation replicates. Each block corresponds to a simulation scenario defined by the total number of predictors (column) and the within-cluster correlation structure (row). The column headings indicate the total number of predictors, and the values in the parentheses indicate the cluster-specific numbers of active/total predictors; the rows represent within-cluster correlation scenarios, with the headings specifying the correlation coefficients for the three clusters. The box colors denote outcome prevalence; within each cell, the results are shown for the two model variants (long.M1 for LCoDaCoRe1 and long.M2 for LCoDaCoRe2), and for the cross-sectional CoDaCoRe applied at each visit (0, 1, 2, 4).

The simulation results are summarized in three sets of boxplots showing the accuracy (Figure 2), sparsity (Figure 3), and FNR (Figure 4), where each set of results is based on 200 replications. In these figures, the columns correspond to the predictor dimensions, headings indicate the total number of predictors, and the values in parentheses indicate the number of active/total predictors per cluster. The rows represent within-cluster correlation scenarios, and the headings specify the correlation coefficients for the three clusters; the box colors denote the outcome prevalence (red = 10%, green = 30%, blue = 50%). Within each cell, the results are shown for the two model variants (long.M1 for LCoDaCoRe1 and long.M2 for LCoDaCoRe2), along with the cross-sectional CoDaCoRe (Gordon-Rodriguez et al., 2022), applied at each visit (0, 1, 2, 4). The visit labels mirror those of the motivating study design (Section 3.2) and are used solely for consistency.

##### 3.1.2.1 Prediction accuracy

As shown in Figure 2, both longitudinal models achieve higher median AUC values than the cross-sectional CoDaCoRe models across all settings. Prediction accuracy does not notably vary across predictor dimensions (columns) but is strongly affected by within-cluster correlations (rows). In particular, the AUC is highest (lowest) when the active predictors are drawn from clusters with high (low) correlations. Variability decreases with increasing case prevalence, where the most stable performance is observed under the balanced outcome scenario (50% prevalence), particularly in the high-dimensional setting with p=200 .

##### 3.1.2.2 Sparsity

The proposed model variants (long.M1 and long.M2) consistently select fewer predictors than the cross-sectional counterparts across all settings (Figure 3). The number of selected variables decreases with increasing problem dimensionality (i.e., columns with p=30,100,200 ), and it is lowest when the active predictors are selected from strongly correlated clusters (top row; ρ=(0.7,0.7,0) ). Case prevalence also influences sparsity, whereby the results are more stable and sparse under the balanced scenario (blue boxes, 50% prevalence) than under the highly imbalanced (red boxes, 10% prevalence) and moderately imbalanced (green boxes, 30% prevalence) settings.

##### 3.1.2.3 FNR

The proposed model variants (long.M1 and long.M2) consistently achieve lower FNRs than the cross-sectional approaches (Figure 4), with the majority of the values being concentrated around 0.05 and fewer extreme-right-tail cases. The FNR declines as the number of predictors increases (i.e., columns with p=30,100,200 ) but increases when the active predictors are selected from strongly correlated clusters (top row; ρ=0.7,0.7,0 ), reflecting the challenge of isolating signals within highly correlated groups. The outcome balance improves detection: under the balanced scenario (blue boxes, 50% prevalence), the proposed model variants exhibit particularly low and stable FNRs, as indicated by their compact boxplots.

### 3.2 Real data application

#### 3.2.1 Data description

The NICHD Fetal Growth Studies - Singleton Cohort(2009–2013) included 2,802 generally healthy women aged 18–40 years with singleton pregnancies at 12 clinical centers in the United States (Grewal et al., 2018; Louis et al., 2015). Within this cohort, a nested case-control subcohort was identified to study gestational diabetes mellitus (GDM), whereby 107 women with incident GDM were identified and matched to two non-GDM controls each based on age ( ± 2 years), race/ethnicity, and gestational age at blood collection ( ± 2 weeks); this procedure yielded plasma samples from 321 participants for our analysis.

The plasma samples were collected over up to four study visits, namely V0 (8–13 gestational weeks (gwks)), V1 (16–22 gwks), V2 (24–29 gwks), and V4 (34–37 gwks), and the exact collection times were recorded. Complete lipidomic data were available at all four visits for all GDM cases and one matched control per case, whereas samples from only visits 0 and 1 were processed for the remaining controls, resulting in an irregularly sampled dataset. Untargeted lipidomic profiling was performed, which resulted in 420 lipid metabolites, including 328 annotated metabolites spanning four major lipid classes: glycerolipids, glycerophospholipids, sphingolipids, and sterol lipids. Detailed descriptions of the laboratory methods and quality control are available elsewhere (Barupal et al., 2018; Cajka et al., 2017), and detailed descriptions of the lipids are reported in Song et al. (2023).

The birth weight and other neonatal anthropometrics were measured within 24 h after delivery, and the LGA measure was defined as birth weight above the 90th percentile based on national standards (Duryea et al., 2014). Accurate prediction of LGA is clinically important because it is associated with increased risk of perinatal complications, such as cesarean delivery, postpartum hemorrhage, and birth trauma (Weissmann-Brenner et al., 2012; Scifres, 2021), in addition to long-term risks such as obesity and chronic diseases (Scifres, 2021; Hong and Lee, 2021). The objective of our analysis was to identify lipid trajectories predictive of LGA through the construction of the log-ratio features from selected subsets of the 328 annotated metabolites.

#### 3.2.2 Analysis results

We applied the proposed method in Algorithm 1 for model estimation and predictor selection. The lipidomic data were first log-transformed, and batch effects were adjusted using the empirical Bayes approach (Johnson et al., 2007). We then performed PACE to project the trajectories onto a truncated eigenspace and retained the first K=4 eigenfunctions, which captured 99.9% of the variance. For regularization, we used the 1-standard-error rule. We reported and interpreted the top-ranked feature identified by LCoDaCoRe and compared it to features from visit-specific analyses to evaluate the predictive performance and selection consistency.

**FIGURE 5:**
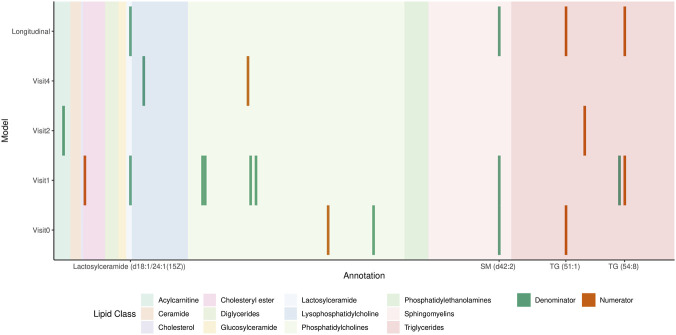
Top-ranked log-ratio features identified by the longitudinal (LCoDaCoRe) model and by the CoDaCoRe models using visit-specific data from the NICHD Fetal Growth Studies. The top row displays results from the longitudinal model, followed by features identified using the cross-sectional CoDaCoRe on data from visits 4, 2, 1, and 0, respectively. The orange and green bars represent the respective components in the numerator and denominator of each log-ratio feature, while the background colors indicate the chemical classes of the lipids. The full names of the lipids are shown along the x-axis only for the feature selected by the longitudinal model.

Figure 5 shows the lipid compositions of the top-ranked feature selected by the proposed longitudinal model (top row) and the features identified by the cross-sectional CoDaCoRe using data from visits 4, 2, 1, and 0 (bottom four rows). Because the visit-specific models treat each visit as a separate cross-sectional snapshot, the selected features may vary across visits. In contrast, the proposed longitudinal model treats each predictor trajectory as a whole to yield consistent feature selection over time. Among all 328 annotated lipids, the most predictive feature included two triglycerides in the numerator and two sphingolipids (i.e., a sphingomyelin and a lactosylceramide) in the denominator, suggesting that these lipid classes have opposing associations with risk of LGA over time. Importantly, all four lipids selected by the longitudinal model were also noted in the visit-specific log-ratio features, primarily those from visits 0 and 1, supporting the longitudinally selected feature choice. Compared to the visit-specific models, the proposed method yielded a sparser set of features.

**FIGURE 6:**
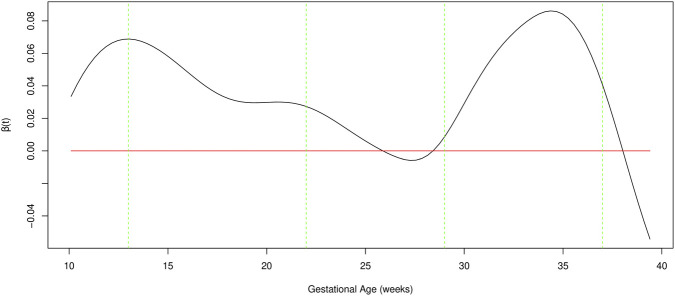
Estimated slope coefficient function of the top-ranked log-ratio feature selected by the longitudinal (LCoDaCoRe) model from the NICHD Fetal Growth Studies data. The vertical green lines indicate the boundaries of the visit windows, while the red horizontal line represents zero.

Figure 6 shows the estimated coefficient function for the log-ratio feature selected by the model for predicting LGA. The function remains generally positive over time, indicating that higher log-ratio values (i.e., higher levels of the two triglycerides relative to the two sphingolipids) are associated with increased risk of LGA. The coefficient value approached zero by approximately 25–30 weeks of gestation (around visit 2), suggesting a weaker association during this period. The effect became more strongly positive again during the late third trimester.

**FIGURE 7:**
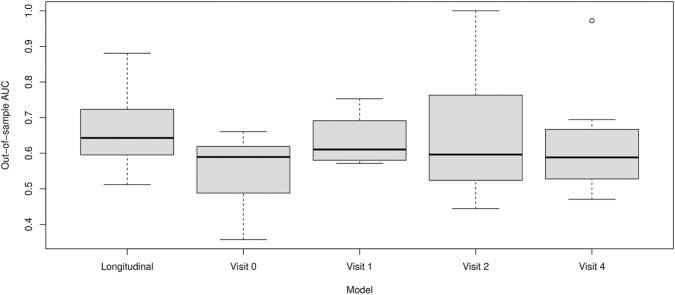
Out-of-sample AUCs from 10-fold cross-validation using data from the NICHD Fetal Growth Studies. The leftmost boxplot shows the results of the longitudinal (LCoDaCoRe) model, while the remaining boxplots show the results of the CoDaCoRe models, which use data from visits 0, 1, 2, and 4 in that order. Higher values indicate better predictive performance for identifying large-for-gestational-age births.

We randomly split the dataset into 10 folds and performed cross-validation using a single fold as the test set and the remaining nine folds as the training set in each iteration. Figure 7 compares the predictive performance of the proposed longitudinal model with that of the cross-sectional models using data from individual visits. The proposed longitudinal method achieved a higher median out-of-sample AUC and exhibited more stable performance across folds, indicating improved predictive performance when incorporating the dynamics of lipidomic patterns.

**FIGURE 8:**
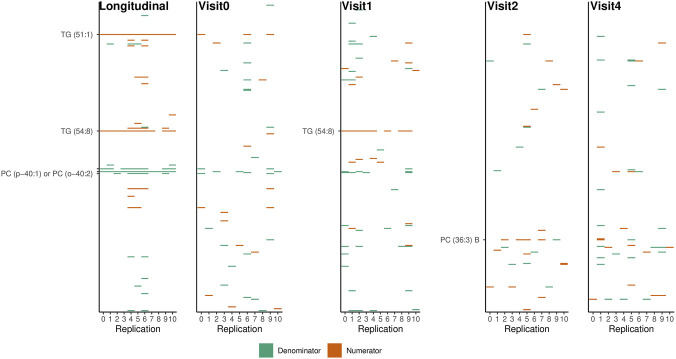
Predictors selected in the top-ranked log-ratio features by the longitudinal (LCoDaCoRe) model and the visit-specific CoDaCoRe models using data from the NICHD Fetal Growth Studies. The data were randomly split into ten folds; in each replication, data from a single fold served as the test set while the remaining nine folds were used as the training set. Replication 0 used the full dataset for model fitting. The orange and green bars indicate predictors in the numerator and denominator, respectively. Lipids selected in at least five of the ten training replications are marked on the y-axis.

To evaluate the stability of variable selection, we compared the top-ranked log-ratio features identified across the ten folds of cross-validation. Figure 8 shows the predictors selected by the LCoDaCoRe and visit-specific CoDaCoRe models. The longitudinal model yielded stable selection results and the same four lipids, as identified by the full dataset, as the top-ranked feature in all training folds. In contrast, the predictors selected by the cross-sectional models varied substantially across folds, resulting in less-robust selection.

## 4 Discussion

In this work, we proposed LCoDaCoRe as a novel supervised learning framework for identifying predictive and interpretable features in longitudinal, high-dimensional compositional data. By unifying compositional data analysis with functional data analysis through log-ratio transformation and principal-component-based trajectory estimation, LCoDaCoRe addresses several key challenges simultaneously, namely the simplex constraint, temporal sparsity and irregularity, and the high dimensionality of predictors. The method is flexible but scalable and yields biologically interpretable log-ratio features, known as balances, that evolve over time and are strongly associated with scalar health outcomes. Our method is motivated by real-world challenges in pregnancy research, where maternal lipid trajectories measured longitudinally during gestation are increasingly relevant for understanding fetal development. In this setting, traditional cross-sectional analyses fail to exploit the temporal structure of the data and are often unstable in terms of variable selection owing to limited sample sizes at each visit. LCoDaCoRe overcomes these limitations by modeling the entire trajectory of each log-ratio feature while incorporating information across time and individuals along with enforcing sparsity through a continuous relaxation strategy.

Simulation studies in various scenarios demonstrate the advantages of LCoDaCoRe in terms of predictive accuracy, parsimony, and false negative control. In particular, its performance remains robust for varying levels of the total number of predictors, predictor correlations, and case prevalence. Compared to cross-sectional CoDaCoRe applied separately to each visit, our longitudinal approach yields more stable and informative features while reducing the risk of overfitting. When applied to lipidomics data from the NICHD Fetal Growth Studies, LCoDaCoRe identified a sparse and biologically plausible log-ratio of two triglycerides to two sphingolipids that showed a consistent association with a risk of LGA births. The selected feature exhibited a time-varying effect that was interpretable and consistent with domain knowledge. Methodologically, LCoDaCoRe contributes to a growing body of literature on learning with compositional and functional data. It extends the CoDaCoRe framework (Gordon-Rodriguez et al., 2022) to handle functional predictors via FPCA (Yao et al., 2005) and scalar-on-function regression (Morris, 2015). The use of continuous relaxation to approximate discrete subset selection allows for a computationally efficient optimization approach using gradient-based methods, even in high-dimensional settings. Additionally, the use of log-ratio balances, as opposed to pairwise log-ratios or amalgamations, ensures interpretability and subcompositional coherence while maintaining flexibility in modeling the non-linear temporal effects.

Several limitations of the proposed method warrant discussion. First, while the method accommodates sparsely and irregularly observed data through PACE, the performance may degrade if the sampling frequency is extremely low or concentrated in narrow time windows. Second, the current implementation uses data-driven eigenfunction bases, which require retraining with new data. Fixed and compactly supported bases, such as wavelets or B-splines, may offer more stable representations and facilitate the identification of time intervals with non-zero effects. Third, the proposed method currently focuses on scalar outcomes, so extension to multivariate or time-to-event outcomes may broaden its applicability in clinical research. Fourth, although the framework accommodates sparse and irregular longitudinal observations, missing values within the observed samples and zero values incompatible with logarithmic transformation are not modeled directly. These feature-level irregularities would need to be handled upstream through some preprocessing steps. Finally, our primary goal is prediction within a fixed dataset for predictor trajectories that are already available, rather than direct deployment of the fitted model to an external dataset. When external validation is desired, a more rigorous strategy would be to re-estimate the shared eigenbasis within each training fold, and then represent the excluded samples using that training-fold basis.

Future work can build on LCoDaCoRe in several directions. From a methodological perspective, the incorporation of hierarchical or group structures among the predictors, such as chemical classes or pathways, could enhance interpretability and the ability to borrow information. From an inferential standpoint, developing formal uncertainty quantifications, such as confidence bands for coefficient functions and stability selection procedures, would support statistical inference. Integrating prior knowledge or covariate adjustment into the balance construction and regression stages may also enable further tailoring of the method to specific biomedical applications. Finally, we applied the model to data from the NICHD Fetal Growth Studies for illustrative purposes; this demonstration is limited to assessing internal discrimination within the sampled set. If generalization to the underlying pregnancy cohort is needed, an extension of our method incorporating the sampling design would be the introduction of inverse-probability weights into the multivariate generalized linear model in Equation 9, for example, through weighted estimation equations or a weighted pseudo-likelihood method (Binder, 1983; Lumley and Scott, 2017). In conclusion, LCoDaCoRe provides a flexible and interpretable approach to analyzing high-dimensional longitudinal compositional data. Its ability to extract dynamic log-ratio features with strong predictive power makes it a valuable tool for biomarker discovery in longitudinal studies with complex data.

## Data Availability

The data analyzed in this study are subject to the following licenses/restrictions: Restrictions apply to the availability of Fetal Growth Study data, which were used with permission for the current study and are not publicly available. However, these data are available upon request and with permission from the National Institute of Childhood and Human Development. Requests to access these datasets should be directed to chenzhe@mail.nih.gov.
